# Perception of non-layperson advisory committee members on the application of a discrete choice experiment instrument to patients and advisory committee members: a qualitative study

**DOI:** 10.1017/S0266462325000029

**Published:** 2025-04-25

**Authors:** Hung Manh Nguyen, Jason Robert Guertin, Daniel Reinharz

**Affiliations:** 1 Département de médecine sociale et préventive, Faculté de médecine, Université Laval, Québec, QC, Canada; 2 Centre de Recherche du CHU de Québec, Université Laval, Québec, QC, Canada

**Keywords:** HTA, committee members, patient and public involvement, discrete choice experiment, decision-making process

## Abstract

**Objectives:**

To explore the view of nonlayperson committee members on the added value of a discrete choice experiment (DCE) instrument to measure patient and committee member preferences for a health intervention.

**Methods:**

Nine semistructured interviews were conducted with voting members from two types of advisory committees in Quebec, Canada: one from the Ministry of Health and Social Services, and eight from the Health Technology Assessment (HTA) agency. The DCE instrument, administrable to patients (i.e., pregnant women) and committee members, was developed and administered to both groups to measure their preferences about the addition of fetal chromosomal anomalies to a prenatal screening program. A conceptual framework consisting of three dimensions (relative advantage, compatibility, and complexity) was used for data collection and analyses.

**Results:**

Committee members considered the DCE instrument, when used with both patients and committee members, to be particularly valuable in raising awareness of potential biases. These biases, generated by committee members’ interests and disciplinary perspectives, can reduce the importance of the patient perspective in decision making by advisory committees.

**Conclusions:**

This qualitative study provides insight into the perceptions of nonlayperson advisory committee members regarding the added value of a DCE instrument administered to patients and committee members regarding an intervention. Additional studies are required to explore the perceptions of other stakeholders (e.g., managers, patients, and public representatives) regarding the application of DCE and to assess its impact on HTA recommendations regarding the value of new health interventions.

## Introduction

In a public healthcare system, considering patient input in the decision-making process regarding services offered to the population improves healthcare service quality (1). In high-income countries, where health technology assessment (HTA) agencies, such as the National Institute for Health and Care Excellence (NICE) in England, All Wales Medicines Strategy Group (AWMSG), and the Canadian Drug Agency (CDA) (formerly known as the Canadian Agency for Drugs and Technologies in Health [CADTH]), are responsible for making recommendations on service provision, the participation of patients and the public across different stages of the HTA process is recommended (2). Finding ways to elicit the opinions of patients and/or the public and supporting their involvement in the deliberative process are central concerns for many HTA agencies (3;4).

Despite the various quantitative and qualitative methods available for eliciting lay opinions from patients or the public in the HTA process, challenges remain (5;6). These challenges include the difficulty of identifying the “right” patients or public members who are representative of the population, interested in the topic, and willing to invest the necessary time and effort to ensure that their group’s views are considered during HTA committee deliberations (7;8). Ensuring that all potential conflicts of interest among selected committee members, including patient representatives, are thoroughly reviewed poses a challenge (6). Difficulties have been emphasized regarding giving voices to patients or representatives of the public in HTA committees, where the main participants are medical professionals, public health specialists, economists, and government officials (9). Patients and public members of these committees may be unfamiliar with the scientific language that typically dominates discussions and promotes evidence-based decision making (10;11). Additionally, committee members representing the scientific side may perceive patients or the public as lacking knowledge and comprehensive perspectives, resulting in the patient perspective not being fully considered (12).

An increase in the number of HTA agencies that are willing to incorporate patient preferences into their assessment of health technologies has recently been observed (13). Among the various preference elicitation techniques, the discrete choice experiment (DCE) is a stated preference method that allows quantitative measurement of preference levels for health interventions (14;15). In a DCE study, a sample of the target population is presented with a series of choice tasks, in which each choice consists of two or three options regarding health interventions. The options consist of a description of the intervention defined through attributes, such as its cost or expected effectiveness, the level of which may vary. For example, Option 1 is less expensive and less effective than Option 2. Respondents in a DCE study should select the option they prefer; therefore, revealing the relative importance they attach to attributes and attribute levels (15). Thus, a DCE study offers HTA committee members quantitative data on the most patient-desirable intervention characteristics and how changes between and within these intervention characteristics influence patient choices.

The DCE method has the potential to address challenges in involving patients and the public in the HTA process, support their discussion with other committee members, and enhance their participation. DCE studies are acknowledged by various HTA bodies, for example, the National Health Care Institute (ZIN) of the Netherlands and the U.K. NICE (16). By offering insights into patient and public perspectives derived from quantitative measurements on a representative sample of the population, the DCE method has the potential to complement other sources of information. This can enhance the dialogue between patient members and other committee members, particularly those whose expertise is more quantitatively focused, thereby strengthening patient participation in the deliberative process (13;17;18).

Most previous DCE studies focused on the value attributed by patients, their relatives, and clinicians to health interventions consumed by patients (19–21). Although the use of patient preference data is not yet routine in some HTA processes, it has been suggested that including preference data could be beneficial in HTA deliberations (13;16). Specifically, it could help assign weights to multiple decision-making criteria, particularly when assessing patient perspectives. Preference data could provide insights complementary to those elicited through other methods, such as patient consultations or patient experience submissions. It could also help understand how individuals with a particular condition make trade-offs between available technologies and identify benefits of a health technology that are not well-captured by clinical or economic evidence.

Currently, interest in the information provided by DCE studies is growing (13), extending beyond patients and the general population to include decision makers and advisory committee members, who also attribute value to interventions likely to be offered within a public health system. However, limited efforts have been made to involve both target groups – patients and advisory committee members – in a DCE using the same instrument to quantitatively measure their preferences for a health intervention (22;23). This application of DCE allows for the collection of patient input on preferences and comparison with those of committee members. Such an approach could enhance the consideration given to patient perspective in the decision-making process and provide data for the citizen representatives in a committee to effectively support their role^1^.

Little is known about how committee members perceive the information provided by a DCE to both patients and committee members, and whether it contributes to ensuring that decisions regarding the appropriateness of introducing interventions into the public healthcare system align with the diverse concerns of stakeholders. Gaining insights from tool users, such as committee members, would guide further research into this use of the DCE approach in decision-making. Therefore, this study aimed to address this gap in the literature by exploring nonlayperson committee members’ perceptions of the expected benefits of data from such a DCE instrument. In this study, the addition of fetal chromosomal anomalies test to a prenatal screening program (i.e., expansion of noninvasive prenatal screening) was used as an illustrative intervention for which the DCE instrument was developed.

## Methods

### How the DCE instrument was developed

The DCE instrument was developed and administered in a project involving both patients and advisory committee members (23;25). The instrument’s seven attributes were identified through consensus reached by pregnant women (as patients) and advisory committee members (policymakers) regarding the provision of a new prenatal screening test to detect chromosomal anomalies (25). The DCE instrument was then administered to representative samples of both the patients (*n* = 272) and committee members (*n* = 24) (23), offering insights into differences in how these groups assessed attributes and attribute levels across various intervention options (e.g., information provided from test results and cost of the test received comparatively less attention from committee members than from the patient group). This qualitative study is a continuation of that research.

### Study design

A qualitative study was conducted using semistructure interviews to explore advisory committee members’ perceptions of the benefits of the DCE instrument in HTA deliberative decision-making. This study involved committee members of the deliberative committees of the Ministry of Health and Social Services in the province of Quebec, Canada. The committees had the mandate to provide recommendations to the Minister of Health and Social Services regarding the appropriateness of offering interventions to the population.

### Conceptual framework

A conceptual framework for use of the DCE instrument as an intervention was employed based on the dimensions of Rogers’ Diffusion of Innovation (DOI) theory (26). These dimensions reflect the characteristics of an intervention (such as new ideas, products, or behaviors) that are evaluated when considering its adoption. The innovation in this study refers to a DCE instrument administered to both patients and committee members. As the end-users, the adoption of this instrument by HTA committee members could be explained by different factors. Furthermore, adoptability is considered here as the ultimate expression of a perception of committee members of the values brought by the application of DCE instrument in HTA process. Therefore, the study conceptual framework is composed of dimensions representing characteristics of innovation, which are expected to be relevant and helpful in reaching the study objective.

**Figure 1.**
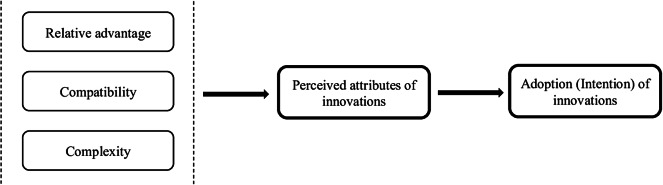
Conceptual framework adapted from Rogers’ Diffusion of Innovation theory conceptual framework.

These dimensions include relative advantage, compatibility, complexity, trialability, and observability (26) are described in Table 1. The last two dimensions (i.e., trialability and observability) were deemed irrelevant to the research question of this study, as they refer to assessing the validity of the DCE instrument. Although these dimensions are relevant to the adoption of the DCE instrument, its validity is evaluated by the HTA methodology teams and subsequently presented to the committee. In this study, the focus was on assessing the adoptability of the data produced by the instrument, as perceived by the committee members. The conceptual framework therefore consists of the three first dimensions of the theory (Figure 1). To construct an interview guide, these dimensions were redefined (see Table 2).

**Table 1.** Five dimensions of Rogers’ Diffusion of Innovation theory

Dimensions	Definitions
Relative advantage	The degree to which a new idea is perceived as superior to the idea it replaces (i.e., an idea that provides unambiguous advantages over the previous approach is more likely to be accepted).
Compatibility	The degree to which a new idea is perceived as consistent with the existing values, past experiences, and needs of potential adopters (i.e., the higher the compatibility of the new idea, the greater the likelihood of its acceptance).
Complexity	The level of difficulty associated with understanding and using a new idea (i.e., simplifying the use of new ideas enhances their likelihood of acceptance).
Trialability	The degree to which a new idea may be experimented with on a limited basis (i.e., new ideas require investing time, energy, and resources. New ideas that can be tried before being fully implemented are more readily adopted)
Observability	The degree to which the results of a new idea are visible to others (i.e., if there are observable positive outcomes from the adoption of a new idea, it will be more likely adopted).

**Table 2.** Conceptual framework’s dimensions

Dimensions	Definitions
Relative advantage	The instrument is perceived as offering added value to HTA committees by enhancing the information provided by other patient and public involvement approaches.
Compatibility	The instrument and information it produces are perceived as compatible with the values, norms, perceived needs, goals, and standard working procedures of an HTA agency.
Complexity	The difficulty in explaining the information provided by the instrument in an HTA agency report is considered acceptable by the HTA committee.

### Sampling and recruitment strategy

The participants of this qualitative study were nonlayperson members of the advisory committees. They were former or current members of two types of provincial advisory committees in Quebec, Canada. The first type consisted of members from the permanent deliberative committees of the provincial HTA agency (*Institut national d’excellence en santé et en services sociaux*) (27). These committees are mandated to evaluate interventions and deliver recommendations to the Quebec Minister of Health and Social Services regarding offering these interventions to the population. The committees are composed of scientists, clinicians, ethicists, managers, and citizens to ensure diverse perspectives are represented in the deliberations. All members hold voting rights on the final recommendations.

The second type comprised members of the Coordination Committee of the Quebec Pre- and Postnatal Screening Programs of the Ministry of Health and Social Services (MSSS), who possess expertise in relevant disciplines, such as geneticists, obstetricians – gynecologists, family physicians, biochemical physicians, midwives, medical technologists, and government managers (28). This committee is responsible for ensuring the standards, quality requirements, and indicators related to the program, providing expert advice, and making recommendations to the MSSS regarding any new screening technology that could be used within the program.

For illustrative purposes, a DCE instrument that had previously been developed for both patients (i.e., pregnant women) and committee members to quantitatively measure their preferences for a prenatal screening test (23;25) was presented during the interviews. Participants were required to have experience in evaluating prenatal screening interventions. This experience was expected to allow participants to understand the composition of the instrument and facilitates discussions about the value of having this tool for their decision-making process.

Given that their opinions were required based on their experiences with a deliberative committee and not as representatives of the HTA, they were identified through official documents. An initial pool of twenty-two potential participants was established, including current and former committee members from both types of committees. A search for their professional email addresses was conducted using official Web site, organizational affiliations, and publications. We were unable to identify contacts for several participants, such as those who had changed their positions. Moreover, participants were purposely sampled to reflect diverse disciplines and a range of attitudes toward DCE studies, based on their full, partial, or nonparticipation in a previous study that administered a DCE instrument to both patients and committee members. The recruitment strategy aimed to increase the sample size until reaching information saturation.

Participants received an invitation via their professional email addresses. The email included a brief introduction to the study’s nature and objectives, with an attached informed consent form providing additional details. The informed consent form stated that participants were identified based on their expertise, their names would be coded for confidentiality, and their answers were personal reflections based on their own experiences, not representing the official position of the committee to which they were members.

Participants were asked whether they would be interested in a half-hour interview to discuss the added value of this DCE instrument. Upon receiving a positive answer, they were contacted to arrange virtual meetings.

### Interview guide

The interview guide was developed based on the three dimensions of the study’s conceptual framework: relative advantage, compatibility, and complexity. The guide was pretested with three members of the HTA committee who were not involved in the study. After the pretest, no modifications were made to the interview guide (Supplementary File 1).

### Data collection

Data collection was conducted from December 2022 to October 2023.

Online meetings were organized using Microsoft Teams at a convenient time for the participants. On the scheduled day, the researchers verbally presented the study and answered any participant questions. Additionally, the researchers provided further clarifications, if needed, before the interviews. Subsequently, participants were asked to confirm their participation and sign an informed consent form.

Semistructured interviews were conducted using the interview guide. Each interview started with a general question regarding the participants’ beliefs regarding the interest of a DCE instrument administrable to both patients and committee members within the work conducted by scientific committees at their HTA agencies. The participants were encouraged to say whatever they wanted without interruption. Additional questions were asked regarding the dimensions of the conceptual framework that had not been discussed previously, depending on the spontaneously generated information and the participants’ capabilities to provide insights into those aspects.

The interviews were recorded with the participants’ agreement and verbatim transcription was performed. The data were securely stored electronically on Université Laval’s server, with access restricted to the research team members.

### Data analysis

Transcripts were imported into NVivo (release 14.23.0, QRS International, 2023) to facilitate data storage and organization for accessibility during the analysis process. Data analyses were independently conducted by two researchers (HMN and DR) using the Framework Method (29;30). Coding was structured based on the dimensions outlined in the conceptual framework. In instances of divergence, a consensus was reached among the researchers.

### Ethical approval

Ethical approval was obtained from the teaching hospital’s ethics committee in Quebec, Canada: *Comité d’éthique de la recherche du CHU de Québec-Université Laval* (project 2020–4877). Signed informed consent was obtained from each participant before the interview.

## Results

A total of sixteen voting members from advisory committees in the province of Quebec were invited to participate, of whom nine consented to be interviewed. All participants were healthcare professionals with expertise in various disciplines, each with more than 5 years of experience in HTA committees. Table 3 summarizes the participants’ characteristics. Even though this study aimed to capture various perspectives, the low response rate questioned whether information saturation was attained.

**Table 3.** Participant characteristics

Characteristics	*N* = 9
Sex	
Male	6
Female	3
Professional background	
Social science	1
Medicine	4
Pharmacy	2
Ethics	1
Biology	1
Previously participated in a DCE study	
Never	4
Invited but did not participate	1
Accepted but did not complete the questionnaire	2
Completed the questionnaire	2

Table 4 presents the three main themes corresponding to this study’s objectives. These themes were initially identified from the data analysis conducted based on the conceptual framework (i.e., relative advantages, compatibility, and complexity). No additional themes emerged from the data analysis with this study topic.

**Table 4.** Summary of themes and added value

Themes	Added values
Relative advantages of a DCE administrable to patients and committee members	Identifying patient perspective for the intervention under HTA evaluation and comparing them with committee members’ perspective
	Supporting patient involvement in the HTA process
Compatibility of DCE in the HTA process	Compatible with HTA decision-making process
	Quantitative data
Committee members’ perceptions of the complexity of DCE	Interpretation of DCE results

### Relative advantages of a DCE administrable to patients and committee members

The participants suggested that a DCE instrument administrable to both patients and committee members could add value to the decision-making process. Specifically, they emphasized that such an instrument could allow the identification of the patient’s perspective, support patient involvement in the HTA process by emphasizing their concerns, and reduce the impact of subjective emotions on the process.

#### Identifying patient perspective for the intervention under HTA evaluation

All participants emphasized a DCE study’s potential to provide supplementary data for identifying patient values that might be affected by the intervention. They asserted that DCE studies allow the production of quantitative data from the patient’s perspective. Although HTA committees often seek patient perspectives on interventions, some committee members emphasized this by stating that, due to the scarcity of data on patient perspectives, results from a DCE study are likely to carry considerable weight, complementing other approaches.

Most participants believed that using a DCE instrument could reduce the risk of bias during information gathering. Two main sources of potential bias were identified among the respondents. First, there is the possibility that the opinions expressed by individuals regarding an intervention under evaluation may not accurately represent the broader group’s perspectives. For example, this situation could have arisen if representatives of the population expressed their personal opinions without referencing the collective view of the population concerning the intervention. Second, some participants, particularly pharmacists, and clinicians, mentioned the risk of selection bias stemming from HTA methodologists, who might have prioritized epidemiological data over lived experience data.

Additionally, some respondents emphasized the potential for bias arising from information collected from patient groups acting as lobbyists or recruited by patient associations or pharmaceutical companies. These committee members tended to assign less importance to such perspectives, particularly during assessments of health technologies.

Using the same DCE instrument for both committee members and patients contributes to a better quantitative understanding of patient perspectives. One participant emphasized that the HTA committee frequently received reports of consultations conducted by HTA staff with patient groups; however, these reports were not systematically presented. Another participant mentioned that patient perspectives are sometimes regarded as new knowledge by scientific experts, making precise valuation challenging. Therefore, generating numerical data from the perspectives of patients and committee members through a DCE instrument may convincingly demonstrate how each group assesses different aspects of an intervention and facilitate the HTA committees’ judgments of patient inputs.

Finally, some participants considered that the DCE method not only diminishes the selection bias of patient participants, which can occur in other approaches, by administering the instrument to a representative group but also provides quantitative data on the importance of intervention dimensions, which have been predefined by the same group of patients.

#### Supporting patient involvement in the HTA process

Two types of benefits were anticipated from the DCE study of patient involvement in the HTA process. First, most participants believed that patient members of the committee could use data provided by the DCE instrument to effectively express their concerns in the same language used by other committee members, particularly doctors, during discussions. Scientific experts often express their perspectives through evidence produced by epidemiological approaches. A concern was raised among committee members that they frequently had to “make do” with data from qualitative studies or consultations involving patients with lived experience. These documents are often lengthy, not organized scientifically, making them difficult to read, and may lack the scientific validity expected by those who often make decisions based on quantitative data.

Second, participants emphasized that the scientific information provided by DCE studies could give more weight to the position of patients and/or public members on HTA committees. They believed that patients often constituted a minority (i.e., only one or two representatives) in deliberative committees, resulting in limited voting influence. Furthermore, committee discussions are typically driven by scientific evidence and require a certain level of scientific understanding, which may pose challenges for patient involvement. Having a well-designed DCE instrument could assist in systematically structuring patient perspectives, similar to how scientists organize their knowledge. This structured approach may enhance the credibility of patient perspectives among all committee members, empowering patient representatives to more effectively advocate their perspectives. Therefore, the DCE method may act as a mediator, fostering a balance within HTA committees of scientific data and patient experience data where scientific dominance is prevalent.

The participants suggested that the greatest added value of using the DCE instrument is that it might prompt committee members to question the possibility that their judgments could be consciously or unconsciously biased by overlooking aspects of the technology that may be less essential to them but are important to patients. One member for example highlighted that some committee members tend to have positive preconceived ideas when evaluating a new technology. Another member expressed concern about the selection of information, noting that scientists might be inclined to disregard letters submitted by patient associations under the assumption that these letters were preformatted by pharmaceutical companies. Hence, they might dismiss a possible opinion of patients, regardless of whether the opinion aligns with the company’s objectives. The participants added that such a DCE instrument can be considered a tool for detecting discrepancies and helping committee members better reflect on their judgments toward an intervention. This is a major concern because although committee members are expected to represent diverse opinions, their recommendations depend on a vote that might not accurately reflect the relative importance of these opinions.

Furthermore, two-thirds of the participants believed that the quantitative measurement of preference scores (i.e., the relative importance of attributes and trade-off estimations) obtained from DCE studies could facilitate their decision-making process in HTA. They perceived that the scores would be particularly valuable when interventions are poorly supported by evidence, such as in the assessment of a promising intervention targeting rare diseases, where the decision cannot be justified solely by epidemiological data. Another participant explained that using a valid instrument, such as the DCE, and its quantitative results on patient preferences and values would assist in reducing the influence of subjective emotionality on the HTA decision-making process.

### Compatibility of DCE in the HTA process

A divergence was evident among participants regarding their perceptions of the compatibility of DCE with the values, norms, perceived needs, and standard procedures of HTA committees.

Most participants agreed that a DCE is compatible with the HTA process owing to its rigorous scientific design. They noted that it allows for the inclusion of dimensions that are important to patients and serves as a systematic alternative to eliciting patients’ perspectives in HTA.

Some participants expressed concerns regarding the instrument that claimed to reflect the multidimensionality of a concept with numerical scores. They believed that it was too complex to be accurately represented by a few simple dimensions defined by a limited number of levels.

This concern applies to the potential benefits of using the same DCE instrument for both patients and committee members. The participants expressed doubts regarding the validity of the concept of a shared measure between two distinct groups, particularly when considering a complex concept such as the attributes of the acceptability of a health intervention. Committee members are expected to hold specific interests and social responsibilities that may differ from those of patients and the public. Even though a DCE instrument fulfills the information requirements within an HTA committee when administered to patients, its relevance to both patients and committee members remains questionable when applied to both stakeholder groups.

Regarding standard procedures in HTA, more than half of the participants considered the DCE method as compatible with the assessment process. Additionally, they emphasized that its compatibility may be dependent on the judgment of HTA professionals – those knowledgeable in methodological approaches and responsible for gathering, analyzing, and synthesizing the necessary information to inform the committee.

### Committee members’ perceptions of the complexity of DCE

Participants’ perceptions concerning the complexity of the DCE method varied. Although some considered interpreting DCE results to be straightforward, particularly for HTA committee members familiar with scientific evidence and epidemiological data, interviews revealed that understanding its complexity might necessitate a fundamental understanding of or previous exposure to DCE.

Committee members who previously participated in a DCE study claimed that the results were easy to use. Others expressed confusion regarding the distinction between constructing a DCE instrument with an attribute-based choice format and understanding how the DCE identifies the relative importance of each attribute. Similar to the compatibility findings, determining the complexity of the method may be viewed as the responsibility of HTA methodologists.

## Discussion

This qualitative study presented the perspectives of committee members from regulatory and HTA agencies regarding the perceived benefits of a DCE instrument administrable to patients and committee members of health technology interventions.

The findings emphasize that participants considered DCE studies a valuable methodological approach for identifying the values assigned by patients to interventions. An important added value for HTA committee members is that a DCE instrument is built with input from the target population, allowing them to identify what is most important for the target population. Its applicability to a representative population sample may add significant value for committee members involved in HTAs.

Moreover, the findings reflect the desire of committee members from regulatory agencies and HTA bodies to support patient involvement in decision-making processes. Several HTA agencies, such as CDA/CADTH in Canada, acknowledged the value of patient involvement in improving the quality and relevance of decisions regarding publicly funded technologies (31;32). Finding a way for patients to be effectively involved in HTA remains challenging (33). Information produced by DCE studies focusing on patient perspectives is informative and considered supportive of the decision-making process, particularly by epidemiologists (16). Integrating this information into the decision-making process may support patients’ voices in HTA committees, where scientific data are often privileged.

Our study indicated that committee members expressed particular interest in the development of DCE instruments for both patient and committee member groups. They viewed the DCE instrument as a valuable tool for highlighting the gap between committee members’ perceptions and the actual incorporation of patient perspectives. Although the information provided by this DCE instrument could support the voice and position of patient members, a similar impact could be expected for other members of the committee, thereby opening the door for further discussion among them. Additionally, the participants acknowledged the risk of subconscious biases generated from committee members’ interests and disciplinary perspectives, which might result in overlooking important patient aspects. Although other studies emphasized the differences in priorities and preferences between committee members and patients (22;34), the impact of those findings remains unclear (35). This qualitative study presents an effort to seek information on the impact of the DCE instrument, particularly on patients and the public’s participation in the HTA decision-making process. Having such a DCE instrument can assist in identifying biases by revealing whether the dimensions important to patients have been overlooked by committee members in their judgment of an intervention. Participants viewed this reflection as helping committee members better fulfill their mandate to provide recommendations for a system accountable to the public. This reflects their ability to fulfill the mandate, which participants perceived as the main advantage of such an instrument.

Previous literature shows significant efforts in developed countries to hear the voices of patients and the public, ensuring that their perspectives have a meaningful impact on the decision-making process related to intervention delivery (31;33). Notably, the International Society for Pharmacoeconomics and Outcomes Research (ISPOR) Task Force recently released a roadmap aimed at enhancing the usefulness and impact of patient preference studies, including DCE, in decision-making (35). The roadmap emphasizes the importance of involving decision-makers in conducting preference studies and understanding how the generated information is received. Aligned with this roadmap, this study provides insights into committee members’ perspectives on the added value of using a DCE instrument for patients and committee members of the intervention. The findings support the rationale for involving committee members at different stages of a DCE study and emphasize the importance of measuring their preferences and comparing them with the preferences of patients during the assessment of an intervention. However, involving committee members in research activities remains a challenge. A robust DCE design requires a substantial sample size to estimate all parameters accurately. Given the small pool of available committee members, the complexity of the DCE design may need to be adjusted, potentially affecting participation rates and limiting the instrument’s ability to capture true preferences. Nevertheless, supporting the use of DCE with patients and committee members presents a particularly impactful aspect for committee members’ judgment in HTA, contributing to efforts to ensure that the involvement of patients and the public plays a more impactful role and that decisions made by committee members reflect the best interests of various stakeholders.

This study had some limitations. The sample size was small and included only nonlayperson committee members (i.e., excluding citizen representatives) who were healthcare professionals. This limits our ability to compare their perceptions of the added value of the DCE instrument with those of other groups represented on the committees, particularly citizen representatives. Advisory committees, whether part of an HTA agency or a regulatory body, typically consist of only a few members. Participants were eligible for our study if they had experience evaluating prenatal screening interventions, which limited the available pool of participants. Because this eligibility criterion was used to facilitate data collection, we cannot rule out the possibility that committee members who were not enrolled in the study may have different perspectives on the added value of the DCE instrument. The refusal rate was high among those approached. Three former committee members who declined participation just mentioned in their responses to our invitation email that they were either retired and no longer interested in research activities or had changed professional positions. Additionally, the study participants had varying levels of familiarity with the DCE method (i.e., participants from the previous DCE survey, including those who completed the study, dropped out, or refused to participate). It is possible that those who refused the interview may have had different views on the DCE instrument.

Another limitation may relate to our use of a conceptual framework inspired by the DOI theory. In our study, we employed only one aspect of the theory: the characteristics of innovation that potential adopters evaluate when deciding whether to adopt an innovation. The focus of the study was on a new application of the DCE approach that involves both patients and committee members in HTA, which is viewed as an innovation, with committee members considered as end-users of the data provided by the DCE instrument. Although concentrating on the characteristics of innovation provided by the theory may help answer our research question, it could limit our ability to uncover other factors influencing committee members’ perceptions of the DCE instrument.

Finally, the generalizability of the findings may be limited to antenatal care and services, as well as to contexts similar to Quebec (Canada), where healthcare interventions are assessed under provincial jurisdiction by an HTA agency independent of the federal agency. Consequently, this study cannot definitively confirm whether information saturation has been achieved.

## Conclusion

This study presents a focused effort to assess the impact of the DCE method on health policymaking by exploring committee members’ perceptions of using a DCE instrument with patients and committee members. This provides evidence supporting the involvement of both key stakeholder groups in the construction and administration of a DCE instrument. Committee members perceived that using the DCE instrument offers added value by increasing awareness among committee members regarding the potential presence of conscious and unconscious biases. This application of the DCE method reduces the extent to which patient and public perspectives are overlooked in the recommendations made by the scientific committees of HTA agencies regarding the value of new health interventions – as illustrated in the case of the addition of fetal chromosomal anomalies to a prenatal screening program.

## Supporting information

Nguyen et al. supplementary material Nguyen et al. supplementary material

## Footnotes

1 DCE is a complex approach that requires significant investment in the development of a comprehensive questionnaire and the analysis of results (24).

## Supplementary material

The supplementary material for this article can be found at http://doi.org/10.1017/S0266462325000029.
