# Supplementary material for: Perception of non-layperson advisory committee members on the application of a discrete choice experiment instrument to patients and advisory committee members: a qualitative study
Source: Int J Technol Assess Health Care. 2025 Apr 25;41(1):e31. doi: 10.1017/S0266462325000029 (PMC12123159; doi:10.1017/S0266462325000029)
Supplement: Nguyen et al. supplementary material [file S0266462325000029sup001.docx]

**PERCEPTION OF NON-LAYPERSON ADVISORY COMMITTEE MEMBERS ON THE APPLICATION OF A DISCRETE CHOICE EXPERIMENT INSTRUMENT TO PATIENTS AND ADVISORY COMMITTEE MEMBERS: A QUALITATIVE STUDY**

**Running title**

Advisory committee members’ perception of DCEs

**Journal**

International Journal of Technology Assessment in Health Care

**Manuscript type**

Assessment

**Authors**

Hung Manh Nguyen, M. D, Ph. D^1^, Jason Robert Guertin, Ph. D^1,2^, Daniel Reinharz, M. D, Ph. D^1^

^1^Département de médecine sociale et préventive, Faculté de médecine, Université Laval, Québec, Canada

^2^Centre de Recherche du CHU de Québec – Université Laval, Québec, Canada

**Corresponding author**

Daniel Reinharz; Tel: +1 418 656-2131 # 406289; E-mail: Daniel.Reinharz@fmed.ulaval.ca

**Present address**

Hung Manh Nguyen, Centre interdisciplinaire de recherche en réadaptation et intégration sociale (CIRRIS), Québec, QC, Canada

**Supplementary file 1: Interview Guide**

*(Please see original French version below)*

**Introduction**

As stated in the consent form, the purpose of this project is to seek the policymakers' perspective on the adoptability of a new methodological approach, called Discrete Choice Experiment (DCE) as a methodology to support the decision-making process. A DCE allows to quantitatively measure the level of preference for an evaluated intervention. The DCE instrument we have developed aims to measure, with the same instrument, the value for the addition of a new prenatal screening test by two target groups: pregnant women and policymakers.

In this study, policymakers are defined as voting members of a scientific committee of a healthcare system such as the *Institut national d’excellence en santé et en services sociaux* (INESSS) or the *Institut national de santé publique du Québec* (INSPQ), whose mandate is to make recommendations to the Minister of Health on the interventions to be offered to the population.

In case you are not very familiar with the DCE approach, here is a brief presentation of what this approach is:

A DCE questionnaire is based on characteristics (dimensions) associated with an intervention being evaluated. These dimensions have been identified in advance by a representative sample of the target population, for example, pregnant women and policymakers involved in decisions about the offer of prenatal services to the population.

An example of a DCE instrument for measuring the value of a prenatal screening test is presented below. This example shows a screening test characterized by seven dimensions, which were defined during the construction of the instrument by the target populations, i.e., pregnant women and policymakers. The dimensions differ according to their levels. Respondents are asked to indicate which option they prefer: Test A, Test B, or neither.

|  | **Test A** | **Test B** |
| --- | --- | --- |
| **Conditions to be screened** | can be treated or lead to a termination of pregnancy | can be treated or lead to termination of pregnancy and should not be rare (less than one in 200,000 individuals) |
| **Information provided to women (The test result that is presented to a pregnant woman is about)** | the risk of disability and its medical implications | the risk of disability |
| **Test performance (degree of accuracy of the test result)** | known | uncertain |
| **Moment at gestational age to obtain the test result** | before or at the second prenatal visit | before or at the third prenatal visit |
| **Degree of test result certainty (to the severity of the disability)** | the child is certain to have a severe physical and/or intellectual disability that will affect the child's quality of life | the child may have the disease. However, having the disease does not necessarily mean that the child will have a severe physical and/or intellectual disability |
| **Test sufficiency** | a positive result can be confirmed during regular prenatal visits | a positive result may require confirmation by tests that are not offered during regular visits |
| **Cost related to the test** | $0 | $400 |
| **Which test would you prefer (pregnant woman)/ Which test would you prefer to be offered to pregnant women during their pregnancy (decision maker)**  Test A Test B Neither | | |

Statistical analysis is then used to determine the value placed on the intervention by the sample.

The purpose of this interview is to understand the added value of such an instrument for perinatal decision-making, according to policymakers. Your participation in the study will help us to better define how the findings from a common DCE instrument for pregnant women and policymakers can contribute to meeting the needs of prenatal screening of the population.

Before we start asking the questions, we would like you to introduce yourself and tell us about your background and experience with prenatal screening programs.

**General question**

The purpose of this exchange is to learn about your interest in an approach in the field of economic evaluations, the DCE, for decisions about the content of the prenatal screening program.

To begin, could you share with us your perspectives on the added value of a common DCE instrument for both patients and policymakers in making decisions about interventions for the population.

**Sub-questions: depending on the information obtained from the general question, some of the following sub-questions may be asked:**

- According to you, are there any benefits of using a common DCE instrument for patients and policymakers (people who have to evaluate the relevance of offering a new test to the population)?
- According to you, are there any disadvantages to using a common DCE instrument for patients and policymakers?
- As a policymaker, which barriers do you see to the acceptability of preference scores produced by a DCE instrument?
- Does such an instrument seem compatible with the process of evaluating an innovation in a deliberative committee of the healthcare system?
- Do you find the complexity of the DCE approach a barrier to its use by a deliberative committee?

**Final question**

Is there anything else we have not addressed that you would like to discuss on this topic?

Thank you for this interview and for your time.

**Annexe 1: guide d’entrevue**

**Introduction**

Tel qu'indiqué dans le formulaire de consentement, ce projet a pour objectif de recueillir l'opinion de décideurs concernant l'adoptabilité d'une nouvelle approche méthodologique, appelée DCE. Le DCE permet de mesurer quantitativement le niveau de préférence pour une intervention évaluée. L'instrument que nous avons développé a pour but de mesurer avec un même instrument, la valeur pour l'addition d'un nouveau test de dépistage prénatal dans 2 groupes cibles : les femmes enceintes et les décideurs.

On entend par « décideurs » dans cette étude, des personnes votantes d'un Comité scientifique d'un organisme du réseau de la santé comme l'INESSS ou l'INSPQ, qui a pour mandat de faire des recommandations au ministre de la Santé, sur les interventions à offrir à la population.

Pour le cas où vous ne seriez pas très familier avec l'approche DCE, voici une brève présentation de ce qu'est cette approche.

Un questionnaire DCE repose sur des caractéristiques (dimensions) associées à une intervention évaluée. Ces dimensions ont été identifiées au préalable par un échantillon représentatif de la population-cible, par exemple, les femmes enceintes et des décideurs impliqués dans les décisions quant à l’offre de services prénataux à la population.

Un exemple d’un instrument DCE pour mesurer la valeur d’un test de dépistage prénatal est présenté ci-dessous. Cet exemple montre un test de dépistage caractérisée par sept dimensions, qui ont été définies lors de la construction de l’instrument par le public cible, soit des femmes enceintes et des décideurs. Les dimensions ont différents niveaux. Les répondants doivent indiquer quelle est l'option qu'ils préfèrent : l’option A, l’option B ou aucune des deux.

|  | **Test A** | **Test B** |
| --- | --- | --- |
| Problème de santé à dépister | peut être traité ou conduire à une interruption de grossesse | peut être traité ou conduire à une interruption de grossesse et ne doit pas être rare (> 1 enfant sur 200 000 naissances) |
| Information fournie sur la base du résultat du test porte sur : | le risque de handicap et les implications médicales du handicap | le risque de handicap |
| Performance du test | connu | incertain |
| Moment d’obtention du résultat du test | avant ou lors de la deuxième visite prénatale | avant ou lors de la troisième visite prénatale |
| Degré de certitude sur le résultat du test quant à la sévérité du handicap | l’enfant aura un handicap physique et/ou intellectuel | l’enfant pourrait avoir la maladie, mais avoir la maladie ne veut pas forcément dire que l'enfant aura un handicap physique et/ou intellectuel sévère |
| Suffisance du test | un résultat positif peut être pris en charge lors de visites prénatales régulières | un résultat positifs pourrait requérir des interventions qui ne sont pas offerts lors de visites régulières |
| Coût du test pour la famille | $0 | $400 |
| **Quel test préféreriez-vous ? (femme enceinte)/ Quel test préférez-vous voir offert aux femmes enceinte lors de leur grossesse ? (décideur)**  Test A Test B Aucun | | |

Une analyse statistique permet par la suite d'établir la valeur accordée par l'échantillon pour l'intervention.

L'entrevue que nous allons avoir vise à comprendre la plus-value d'un tel instrument pour la prise de décision en périnatalité, selon des décideurs. Votre participation à la recherche nous aidera à mieux définir comment les données probantes issues d'un instrument DCE commun aux femmes enceintes et aux décideurs, peuvent contribuer à répondre aux besoins de dépistage prénatal dans la population

Avant de vous poser les questions, nous souhaiterions que vous vous présentiez en nous disant quelle est votre formation, et que vous nous parliez de votre expérience avec les programmes de dépistage prénatal.

**Question générale**

Cet échange vise à connaître votre intérêt pour une approche dans le champ des évaluations économiques, le DCE, pour les décisions sur le contenu du programme de dépistage prénatal.

Pour débuter, pourriez-vous nous partager vos idées sur la plus-value d'un instrument DCE commun à des patients et à des décideurs, pour la prise de décision sur les interventions à offrir à la population.

**Sous-questions : en fonction de l'information produite grâce à la question générale, certaines des sous-questions ci-dessous pourront être posées**

- Selon vous, est-ce que l’utilisation d'un instrument DCE commun aux patients et à des décideurs (des personnes qui ont à évaluer la pertinence d'offrir un nouveau test à la population), présente des avantages ?
- Selon vous, est-ce que l’utilisation d'un instrument DCE commun aux patients et à des décideurs, présente des désavantages ?
- Quels sont les obstacles que vous envisagez à l'acceptabilité par les décideurs, de scores de préférence produits avec un instrument DCE ?
- Est-ce qu'un tel instrument vous parait compatible avec le processus d'évaluation d'une innovation dans un comité délibératif du réseau de la santé ?
- Est-ce que la complexité de l'approche DCE vous semble une barrière à son utilisation par un comité délibératif ?

**Dernière question**

Est-ce qu’il y a d’autres aspects que nous n’avons pas abordés et dont vous aimeriez discuter à ce sujet ?

Je vous remercie pour cet entretien et pour le temps que vous m’avez accordé.
